# Consumptives’ Home

**Published:** 1871-04

**Authors:** 


### CONSUMPTIVES' HOME.

In the long list of charities sustained by the abounding liberality of New Yorkers, one of the most praiseworthy is a dispensary for diseases of the throat and lungs at 234 Fifth Street, New York City. Dr. A. Ruppner is the managing surgeon. During 1870, two hundred and sixty-four persons applied for gratuitous aid, not one of whom was sent empty away. If any of our readers have it in their hearts to lend a helping hand to so deserving a charity, they can remit in the form of a check or post-office order, payable to the order of "Hon. E. Ward, President," or Col. Rush C. Hawkins, 234 Fifth Street,

Ne ork City; and the interest of humanity may be subserved by bearing the address in mind, in case they should come across persons who needed aid in this direction.

